# Analysis of Multiple HPV E6 PDZ Interactions Defines Type-Specific PDZ Fingerprints That Predict Oncogenic Potential

**DOI:** 10.1371/journal.ppat.1005766

**Published:** 2016-08-02

**Authors:** Miranda Thomas, Michael P. Myers, Paola Massimi, Corrado Guarnaccia, Lawrence Banks

**Affiliations:** 1 Tumour Virology, International Centre for Genetic Engineering and Biotechnology (I.C.G.E.B.), Trieste, Italy; 2 Protein Networks, International Centre for Genetic Engineering and Biotechnology (I.C.G.E.B.), Trieste, Italy; 3 Biotechnology Development, International Centre for Genetic Engineering and Biotechnology (I.C.G.E.B.), Trieste, Italy; University of Wisconsin Madison School of Medicine and Public Health, UNITED STATES

## Abstract

The high-risk Human Papillomavirus (HPV) E6 oncoproteins are characterised by the presence of a class I PDZ-binding motif (PBM) on their extreme carboxy termini. The PBM is present on the E6 proteins derived from all cancer-causing HPV types, but can also be found on some related non-cancer-causing E6 proteins. We have therefore been interested in investigating the potential functional differences between these different E6 PBMs. Using an unbiased proteomic approach in keratinocytes, we have directly compared the interaction profiles of these different PBMs. This has allowed us to identify the potential PDZ target fingerprints of the E6 PBMs from 7 different cancer-causing HPV types, from 3 HPV types with weak cancer association, and from one benign HPV type that possesses an ancestral PBM. We demonstrate a striking increase in the number of potential PDZ targets bound by each E6 PBM as cancer-causing potential increases, and show that the HPV-16 and HPV-18 PBMs have the most flexibility in their PDZ target selection. Furthermore, the specific interaction with hScrib correlates directly with increased oncogenic potential. In contrast, hDlg is bound equally well by all the HPV E6 PBMs analysed, indicating that this is an evolutionarily conserved interaction, and was most likely one of the original E6 PBM target proteins that was important for the occupation of a potential new niche. Finally, we present evidence that the cell junction components ZO-2 and β-2 syntrophin are novel PDZ domain–containing targets of a subset of high-risk HPV types.

## Author Summary

The cancer-causing Human Papillomavirus (HPV) E6 oncoproteins have a unique carboxy terminal PDZ binding motif (PBM), which interacts with a number of different cellular PDZ domain-containing substrates. The PBM has important functions in both the viral life cycle and in HPV-induced malignancy. In this study we have used a proteomic approach to compare the ability of multiple HPV E6 oncoproteins to interact with different cellular PDZ proteins. We show a striking increase in the number of PDZ proteins recognised as the oncogenic potential of the individual E6 increases. Furthermore, we define combinations of PDZ proteins that are predictors of oncogenic potential, whilst others represent evolutionarily conserved targets bound across the evolutionary spectrum of low and high-risk PBM-containing E6 proteins. Taken together, these studies shed light on the functional conservation in the E6 PBM across multiple HPV types and support the hypothesis that ancestral PBM evolution originally conferred association with a restricted number of PDZ targets, which has, over time, evolved, to provide increased levels of flexibility and hence increase the number of cellular PDZ partners that can be bound by the cancer-causing E6 oncoproteins.

## Introduction

High-risk human alpha-papillomaviruses are the causative agents of cervical cancer, other anogenital cancers, and an increasing proportion of head-and-neck cancers [1,2]. They express two major oncoproteins, E6 and E7, which are essential for maintenance of the transformed phenotype, and blocking their expression results in cessation of tumour growth, with senescence or apoptosis of the tumour cells and derived cell lines [3]. This reflects their interaction with vital cellular pathways, with E7 stimulating cell-cycle progression, at least in part through its targeting of the pRB pocket proteins [4,5], while E6 acts to inhibit apoptosis through its targeting of p53 [6] and Bak [7,8].

Despite the critical importance of targeting p53 for E6's oncogenic activity, it is clear that other functions of E6 also play essential roles during tumour formation. One such activity is the ability to bind proteins containing PDZ domains. All the high-risk HPV types contain a Class I PDZ (PDS95/Dlg/ZO) binding motif (PBM) at the extreme carboxy terminus of E6, which is absent, or aberrant in the majority of low-risk types (the Papillomavirus Episteme, PaVE, http://pave.niaid.nih.gov/). The HPV E6 PBM plays a role in multiple E6 functions. It contributes directly towards increasing proliferation in the infected epithelium, and its loss results in a decrease of viral genome amplification and ultimately in loss of viral episomes [9,10,11,12,13]. In addition, it plays an important role in the ability of E6 to induce characteristics of cell transformation in various tissue culture models, and plays a role in the ability of E6 to cooperate with E7 in the induction of tumours in transgenic mice [14,15,9,16].

Many PDZ domain-containing proteins have multiple protein interaction domains, allowing them to act as nodes controlling various cellular processes whose disruption can contribute to malignant transformation [17]. The PBM gives E6 the potential to interact with a discrete set of cellular proteins–those containing PDZ domains—and approximately 12 PDZ-containing proteins have been proposed as *bona fide* E6 binding partners [3,18,19] although the isolated PDZ domains of many more proteins appear to be capable of binding to the E6 PBM [20,21,22]. Previous work indicated that there are likely to be differences in the spectrum of PDZ-containing binding partners of E6, depending on differences in the protein sequence upstream of the canonical PBM, as well as differences in the non-conserved residues within it [20,23,24].

Despite a large body of data reporting multiple potential PDZ-binding partners of HPV-16 and HPV-18 E6, there have been no comparisons with other HPV types, and in particular with those from different categories of known cancer risk. For example, HPV-16 and HPV-18 can be considered to be Group 1 cancer-causing, while HPV-66 is very rarely associated with cancer and is considered to be Group 2B, and HPV-40 is never found in cancers and is classed as Group 3. Indeed HPV-40 E6 has recently been described as having an ancestral prototype class I PBM [25]. Therefore we have been interested in investigating whether the subtle differences in the different E6 PBMs are also reflected in differences in substrate selection. In addition, we also wanted to determine whether any of these substrate fingerprints, potentially unique for each HPV E6 type, might shed light on the key players required for oncogenic potential and, conversely, represent evolutionarily essential interactions that originally evolved to facilitate new niche-colonising characteristics.

To investigate this we have performed an unbiased proteomic analysis in keratinocytes of the PDZ target specificities of a number of high-risk HPV E6 proteins. The results show that hDlg is a major PDZ-containing target of all the PBM-containing HPV E6 proteins analysed, regardless of their oncogenic potential. In contrast, differences in the selection of other PDZ-containing targets, both in their identities and in the numbers of different proteins bound, correlate closely with the degree of cancer-association reported for the various HPV types.

## Results

### Cancer-causing HPV E6 PBMs display broader substrate specificity than non-cancer-causing E6 PBMs

**Fig 1:**
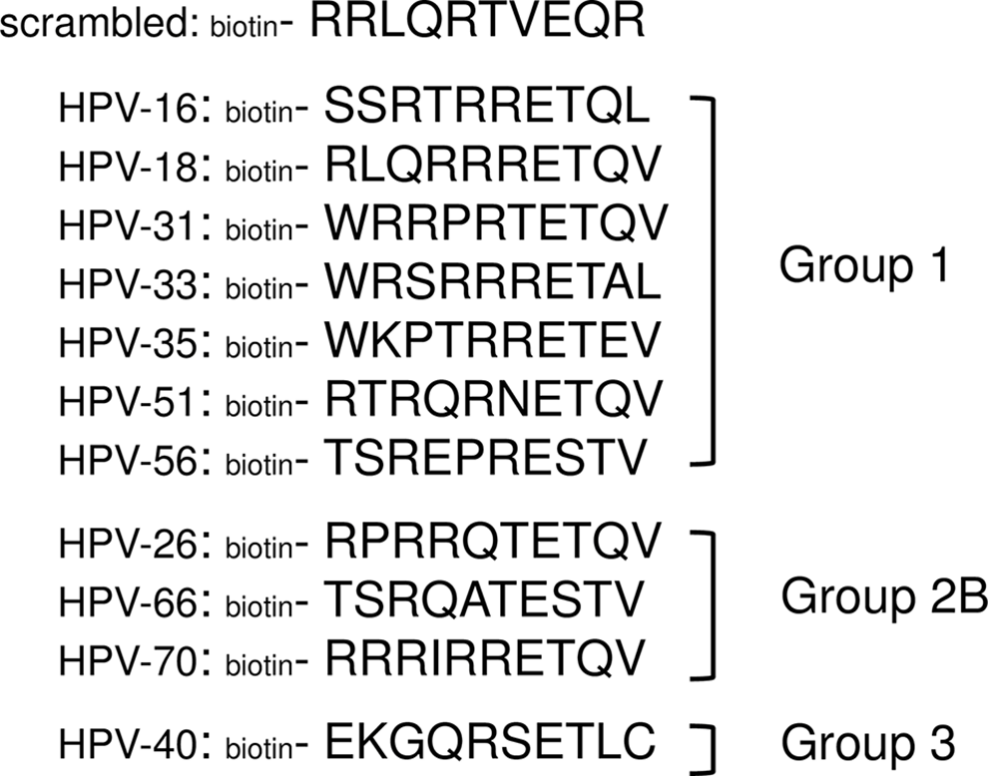
The biotinylated peptides used in this study; the peptides correspond to the 10 C-terminal amino acid residues from the E6 protein of each virus type. The scrambled peptide has the same amino acid composition as the HPV-18 peptide. Group 1 HPV types are defined as *definitely* carcinogenic in humans; Group 2B as *possibly* carcinogenic in humans, and Group 3 as no risk (IARC Monograph 100B).

Previous work had shown that up to 9 amino acid residues upstream of the C-terminus could affect the PDZ protein selectivity of the E6 PBM [20,23,26]. We therefore synthesised peptides corresponding to the ten C-terminal amino acid residues of E6 proteins from HPV-16, -18, -31, -33, -35, -51, and -56, from high-risk group 1 viruses, all of which are defined as carcinogenic in humans [1]. We also synthesised similar peptides from HPV-26, -66, and -70 E6s, from high-risk group 2B viruses that are defined as being possibly carcinogenic in humans [1]. We also included the C-terminal peptide from HPV-40 E6, which has no cancer-risk and has recently been shown to possess an ancestral PBM [25]. These HPV types were selected to represent a wide variety of the E6 C-termini sequences, and these, as well as that of the scrambled Control peptide, are shown in Fig 1.

These peptides, conjugated to magnetic streptavidin beads, were incubated with extracts of HaCaT cells, as described in Methods, and the bound cellular PDZ proteins were identified by mass spectroscopy. The results discussed below are the pooled results obtained from at least two independent pull-downs for each peptide. No PDZ domain-containing proteins were pulled down by the scrambled peptide in any of the assays.

**Fig 2:**
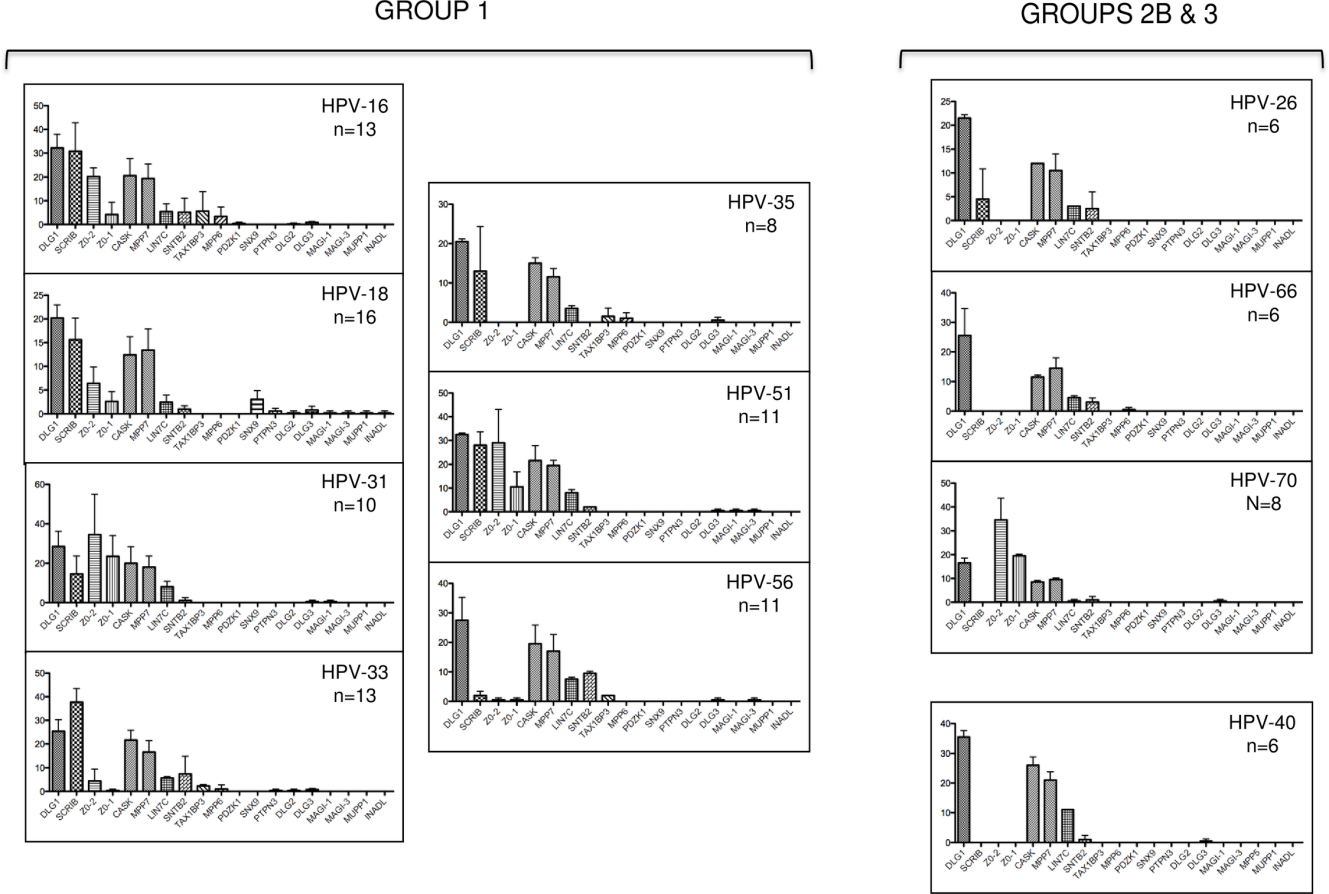
Peptide pulldown assays analysed by mass spectroscopy. Mass spectroscopy results of the pulldown assays from HaCat cells. Each histogram shows the numbers of peptides from each cellular PDZ domain–containing protein that was pulled down from HaCat extracts; n = total number of PDZ proteins pulled down by the E6 C-terminal peptide from HPV types 16, 18, 31, 33, 35, 51, 56 (Group 1); HPV types 26, 66, 70 (Group 2B); HPV type 40 (Group 3). The histograms were drawn using the Prism program, error bars show Standard error of the Mean. The scrambled peptide pulled down no PDZ domain-containing proteins in any assay.

**Fig 3:**
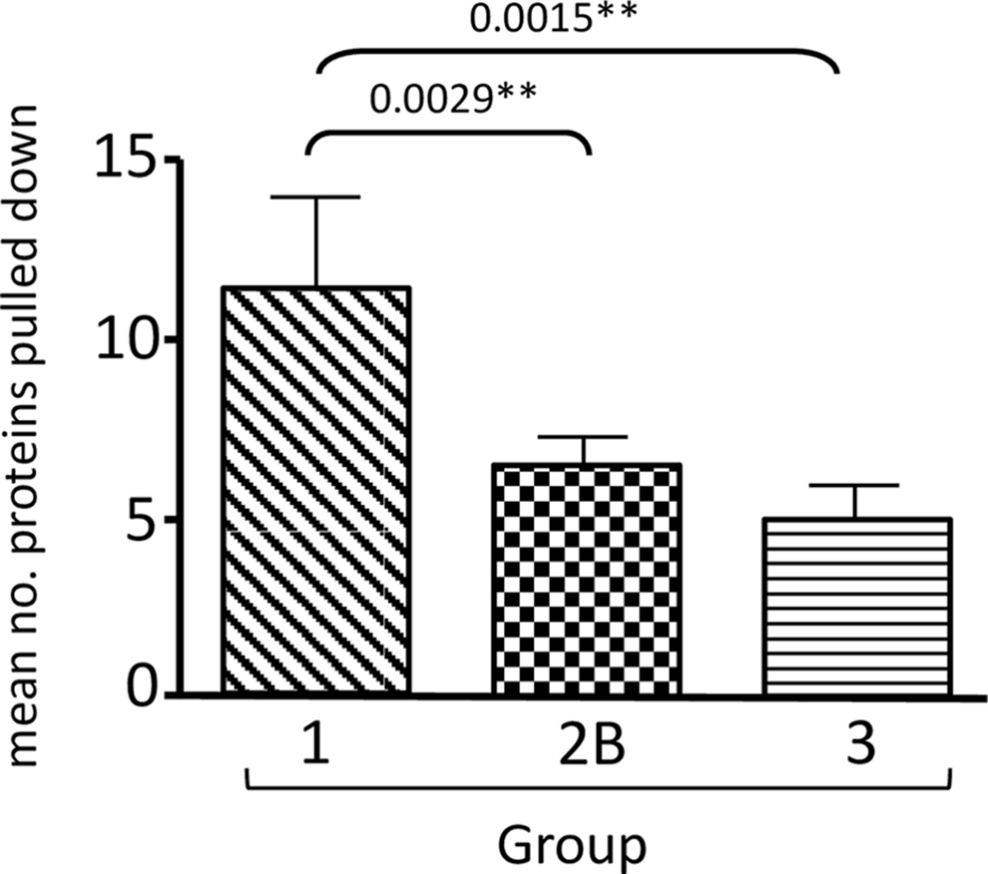
The mean numbers of PDZ domain-containing proteins pulled down by peptides from the Group 1 HPV types was significantly greater than the number pulled down by Group 2B (P = 0.0029) or Group 3 (P = 0.0015) types. Numbers were analysed using the Prism software and standard deviations are shown. The scrambled peptide pulled down no PDZ domain-containing proteins in any assay.

The histograms in Fig 2 show a summary of the mean numbers of peptides from the different PDZ domain-containing proteins pulled down by each E6 C-terminus. As can be seen, a total of 19 different PDZ domain-containing proteins were identified as being potential interacting partners of the different HPV E6 peptides analysed, a number that compares favourably with those reported in other analyses [21,22]. However, it is also clear that no single E6 peptide interacts with all of the PDZ proteins detected, suggesting that each HPV E6 type has its own spectrum of PDZ domain-containing targets, although the HPV-18 E6 PBM appears to have the broadest substrate specificity. It was possible that differences in E6 peptide solubility, in the concentration of the peptides bound to the beads, or in bead loss during the purification might influence the numbers of PDZ domain-containing proteins precipitated by each E6 C-terminal peptide. However, when these data were normalised to the numbers of DLG1 peptides precipitated, no material differences were seen in the binding profiles (S1 Fig). Perhaps more importantly, examination of the numbers of PDZ-containing proteins bound by each peptide (Fig 3) showed that the mean number of proteins bound by all the Group 1 peptides was significantly higher than the number bound by the Group 2B (P = 0.0029) and the Group 3 peptides (P = 0.0015).

**Fig 4:**
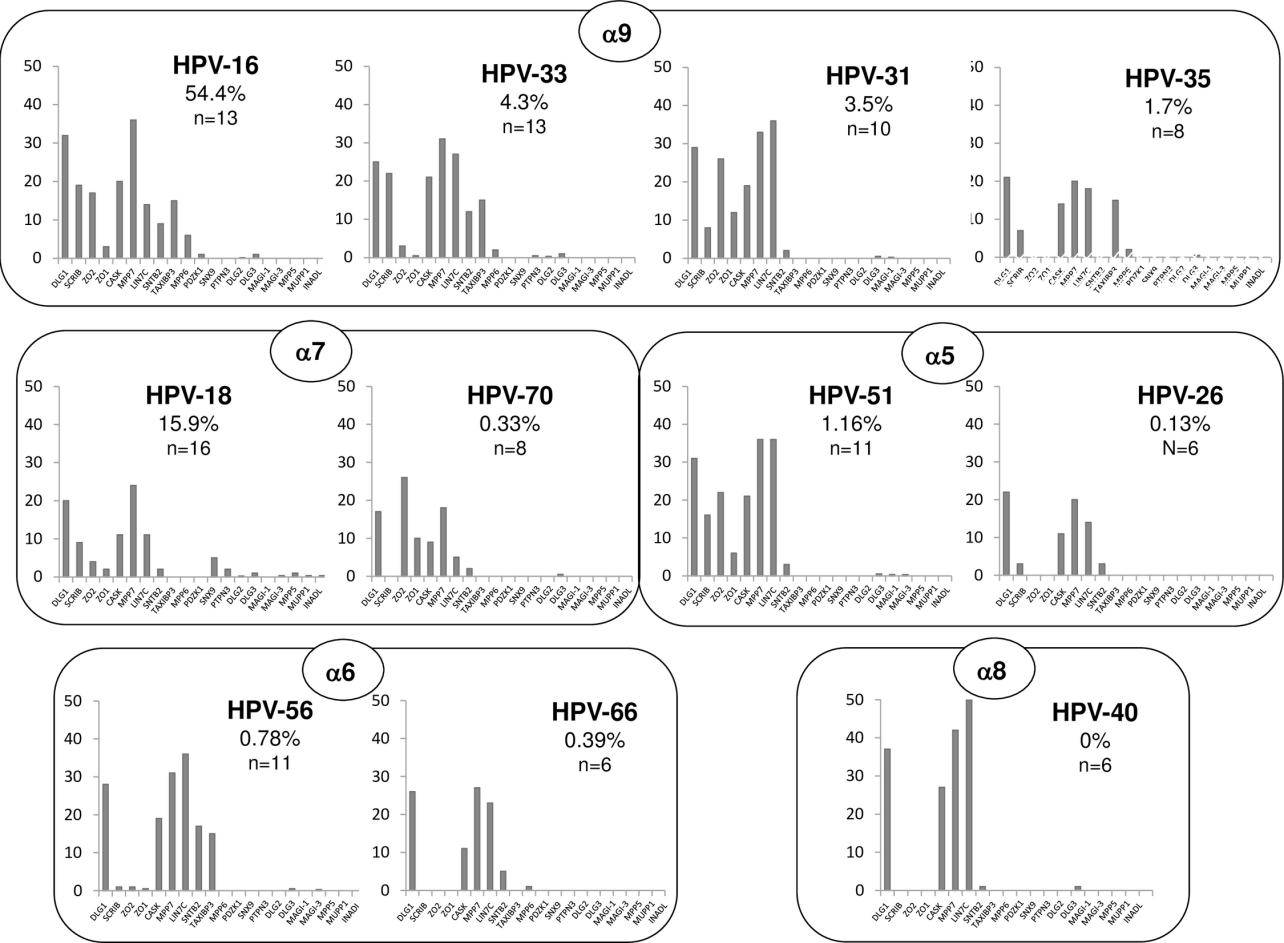
The number of cellular PDZ proteins targeted by the E6 PBMs increases with the cancer-causing potential of the virus type. The histograms show the number of PDZ domain-containing protein peptides pulled down by each E6 PBM, adjusted for the molecular weight of the protein concerned. The histograms are arranged by HPV phylogenetic group (boxes) and by the percentage of total invasive cervical cancers associated with each HPV type (highest on the left); the percentages are shown.

This trend is confirmed in Fig 4, where the histograms of mean peptide number, corrected for protein molecular weight, are grouped by HPV phylogeny, in order of cancer association (highest on the left). This analysis shows first that when the molecular weight of the PDZ protein is also taken into consideration this does not unduly affect the overall comparative interaction profiles of the different E6 proteins. Perhaps more importantly, these results also show that, within each phylogenetic group, the more strongly an HPV type is associated with cancer, the broader the range of PDZ proteins bound by its PBM. Similarly, the numbers of peptides bound increases correspondingly; for example, in the case of the α6 phylogenetic group, the Group 1 HPV-56 PBM interacts with a broader spectrum of PDZ domain-containing proteins than the Group 2B HPV-66 PBM, and binds them to a higher degree.

**Fig 5:**
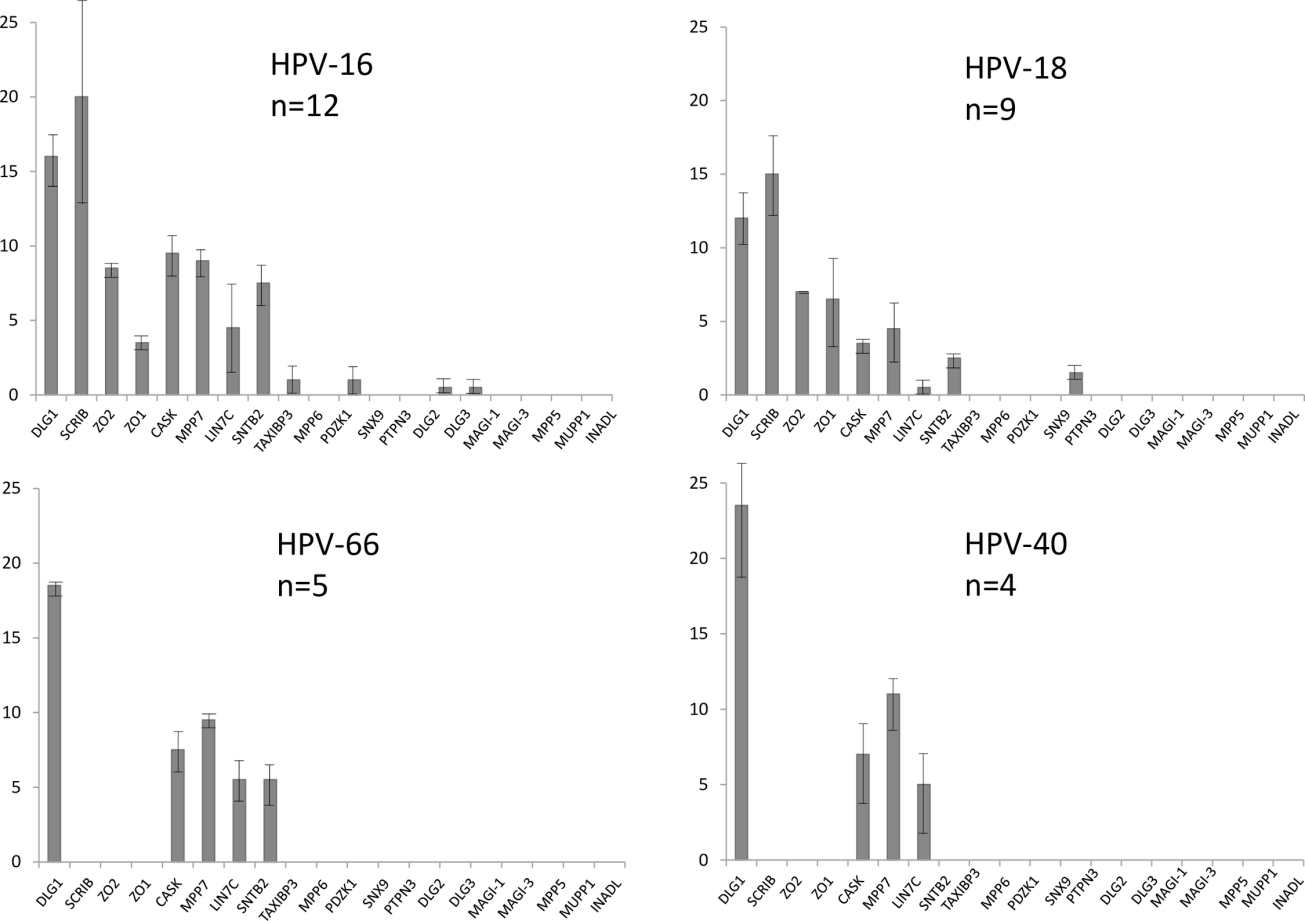
Mass spectroscopy results of the pulldown assays from Normal Immortal Keratinocytes (NIKS), using E6 C-terminal peptides from HPV types representing Group 1 (HPV-16, HPV-18), Group 2 (HPV-66) and Group 3 (HPV-40). The histograms show the numbers of peptides from each cellular PDZ domain–containing protein that was pulled down from NIKS cell extracts; n = total number of PDZ proteins pulled down by each E6 C-terminal peptide. The histograms were analysed using the Prism program and standard deviations are shown. The scrambled peptide pulled down no PDZ domain-containing proteins in any assay.

To confirm that this trend is more generally applicable, and not specific only to HaCat cells, we then performed pulldowns using extracts of normal immortal keratinocytes (NIKS) and peptides corresponding to the C-termini of HPV-16, HPV-18, HPV-66 and HPV-40 (being representative of Groups 1, 2 and 3, respectively). The results obtained from mass spectroscopy analysis of two separate experiments are shown in Fig 5, where it can be seen that HPV-16 and HPV-18 E6 C-terminal peptides again interact with a broader spectrum of PDZ-containing substrates than C-terminal peptides from HPV-66, which in turn binds more than the HPV-40 PBM. It is also clear that all the E6 PBM peptides bind to hDlg1, but that only the peptides corresponding to Group1 E6 PBMs interact with Scrib or ZO-2. The higher the percentage of cancers, the higher the likelihood of binding both proteins, while the HPV-40 E6 binds neither.

### hDlg1 is a common target of the HPV E6 PBM, regardless of transforming potential

**Fig 6:**
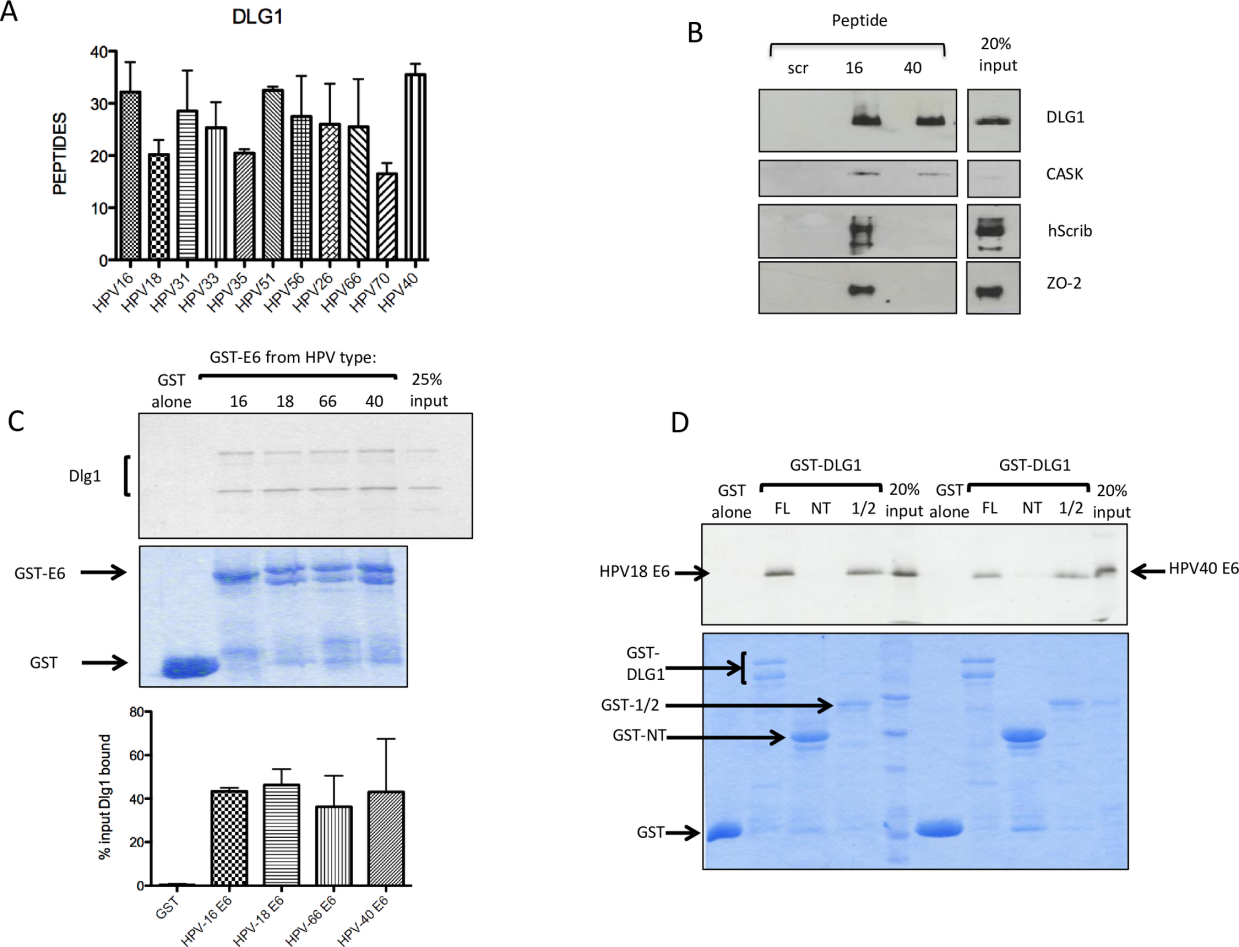
DLG1 is a common target of the E6 proteins, regardless of oncogenic potential. **A.** DLG1 binds to the E6 PBMs of both oncogenic and non-oncogenic HPV types; the histogram shows mean peptide numbers from the mass spectroscopy analysis of all pulldowns, analysed using Prism software, error bars show Standard Error of the Mean. **B.** The non-oncogenic HPV-40 E6 PBM binds DLG1 to a level similar to the HPV-16 E6 PBM. A peptide pulldown from HaCat cell extract, using the control (scr), HPV-16 and HPV-40 peptides, was analysed by western blot and probed for DLG1, CASK, hScrib and ZO-2, as indicated. **C.** DLG1 binds to the E6 proteins of both oncogenic and non-oncogenic HPV types. In vitro translated radiolabelled DLG1 was incubated with the full-length HPV-66 and HPV-40 E6 proteins expressed as GST fusion proteins. GST alone, GST-HPV-16E6 and HPV-18-E6 were included as controls. The upper panel shows the autoradiograph of bound DLG1 and the lower panel shows the Coomassie stained gel. Arrows indicate the GST-E6 proteins. The histogram shows the quantitation of at least three binding assays, analysed by the ImageJ and Prism programmes. Standard deviations are shown. **D.** The non-oncogenic HPV-40 E6 protein binds to DLG1 via its PDZ domains. A GST-pulldown assay, using in vitro translated radiolabelled HPV-18 and HPV-40 E6 proteins, together with GST fusion proteins with full-length DLG1(FL), the N-terminus of DLG1, lacking any PDZ domain (NT), and the first 401 amino acid residues of DLG1, containing the PDZ domains 1 and 2 (1/2). The upper panel shows the autoradiograph of the bound E6 proteins, the lower panel shows the Coomassie stained gel. Arrows indicate the GST fusion proteins.

It has long been clear that the HPV-16 and HPV-18 E6 proteins interact with hDlg1 in a PBM-dependent manner [14,27,28], and it was suggested that such interactions might be important for transforming potential. However recent studies [25] have shown that the E6 proteins of the benign α8 HPV group (HPV types 7, 40, 43 and 91) have a primordial type 1 PBM (-ETxC), and we were therefore interested in comparing the profile of PDZ proteins bound by the HPV-40 E6 PBM with those obtained from the high-risk virus types. As was seen in Fig 2, hDlg1 was the most conserved of the PDZ domain-containing proteins bound by the different HPV E6 C-terminal peptides. In fact, the HPV-40 E6 PBM appeared to have one of the highest affinities for hDlg1: as can be seen from Fig 6A and 6B, it bound hDlg1 to a level similar to that seen with HPV-16 E6. It is also interesting to note that the hDlg1-associated proteins, MPP7, Lin7c and CASK, are also present in all of these interaction assays, indicating that the different E6 peptides can bind the whole hDlg1 complex.

To confirm that the full-length E6 proteins indeed interact with hDlg1, we expressed the E6 proteins of HPV-16 and HPV-18 (Group 1), HPV-66 (Group 2B) and HPV-40 (Group 3) as GST fusion proteins and performed GST pulldown assays with in vitro translated radiolabelled hDlg1. It is clear from the representative autoradiograph and the quantitation that HPV-66 and HPV-40 E6s bind Dlg1 equally as well as the E6 proteins of HPV-16 and HPV-18 (Fig 6C).

The HPV-40 E6 PBM (ETLC) is quite distinct from the canonical type 1 PBM (xT/SxV/L), but it is still, nonetheless, defined as a potential class I motif [29]. However, in order to determine whether it does indeed recognise hDlg1 in a manner similar to HPV-18 E6, we performed pulldown assays using in vitro translated radiolabelled HPV-40 E6 protein and GST fusions with the hDlg1 full-length protein, with the N-terminus of DLG1 (containing no PDZ domains), and with the N-terminus plus the first two PDZ domains of DLG1, including HPV-18 E6 as control. From Fig 6D it can clearly be seen that the HPV-40 E6 protein binds to GST-hDlg1 through its PDZ domains, in a manner similar to the HPV-18 E6 protein, confirming a previous report [25] that the primordial PBM of HPV-40 E6, although unconventional, is indeed functional.

These results suggest that hDlg1 is an evolutionarily highly conserved target of these different HPV E6 oncoproteins. It also suggests that the ability to interact with DLG1 is, alone, independent of the ability of the viruses to cause cancer, and most likely represents a very early evolutionary adaptation during the development of the HPV E6 PBM.

### hScrib is preferentially bound by cancer-causing HPV E6 oncoproteins

**Fig 7:**
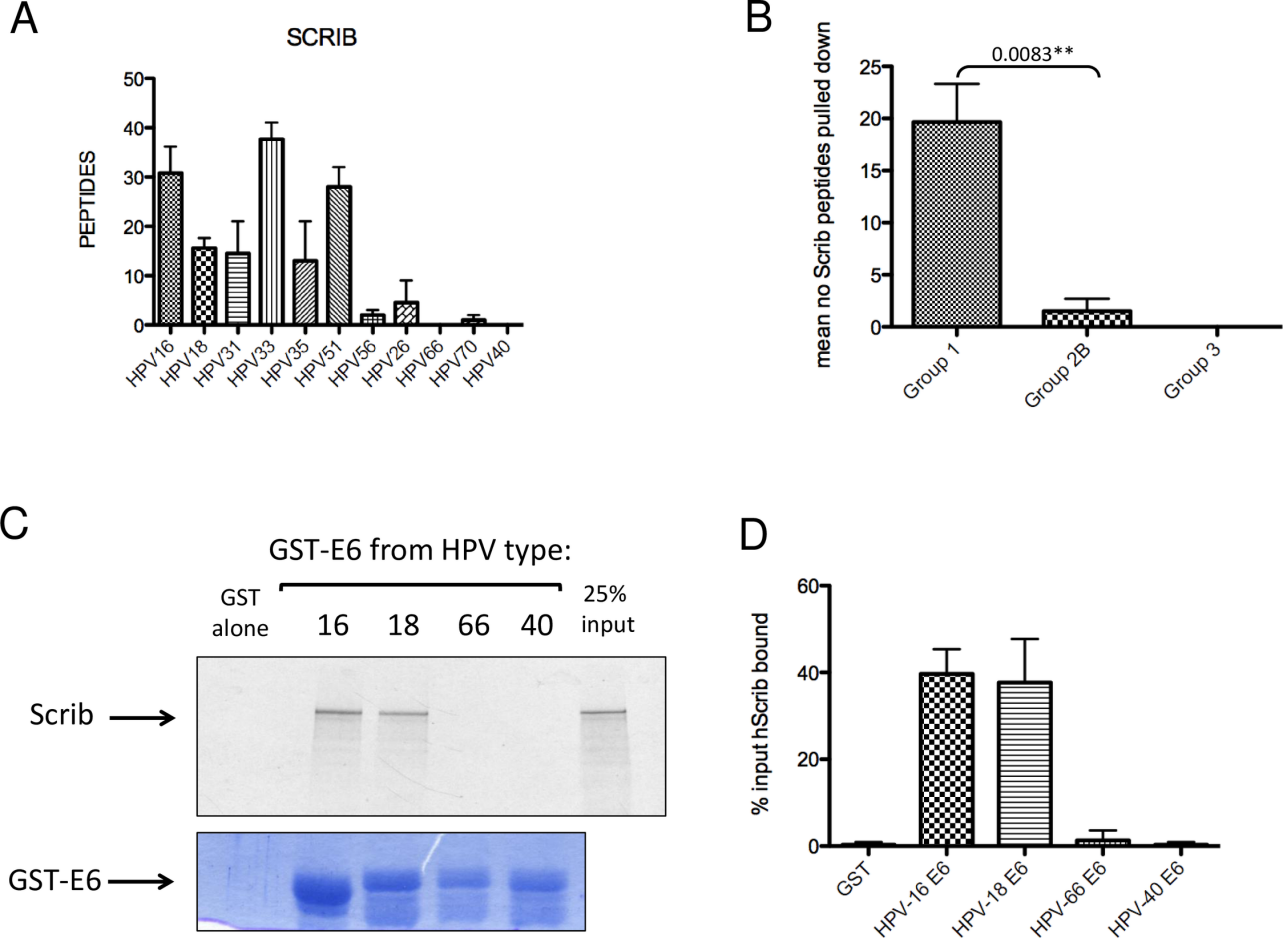
hScrib is preferentially bound by cancer-causing HPV E6 proteins. **A.** The histogram shows the mean numbers of hScrib peptides pulled down by each E6 C-terminus peptide, detected by mass spectroscopy. Standard Errors of the Mean are shown. **B.** Comparison of the numbers of hScrib peptides pulled down by the Group 1 and Group 2B peptides shows a statistically significant (P = 0.0083) difference. Numbers were analysed by Prism software and standard deviations are shown. **C.** hScrib is bound by the E6 proteins of oncogenic HPV types. In vitro translated radiolabelled hScrib was incubated with the full-length GST-HPV-16E6, HPV-18-E6, HPV-66-E6 and HPV-40-E6 proteins. The upper panel shows the autoradiograph of bound hScrib and the lower panel shows the Coomassie stained gel. Arrows indicate the GST-E6 proteins; the GST alone is not visible on a gel of low enough percentage acrylamide to allow hScrib visualisation, but was equivalent to that in Fig 2C and 2D. **D.** The histogram shows the quantitation of at least three binding assays, analysed by the Image J and Prism programmes. Standard deviations are shown.

hDlg1 and hScrib are components of the Scribble polarity complex, which, amongst other functions, defines the adherens junctions (AJs) between cells. Paradoxically, hScrib is also a target of HPV-16 and HPV-18 E6 [30], although previous studies have indicated subtle differences between the different HPV types in their preferences for hDlg1 or hScrib [31]. The numbers of hScrib peptides pulled down by the various HPV E6 C-terminal peptides is shown in Fig 7A, where it is clear that there is much greater variability in the degree of hScrib interaction compared with that of hDlg1. It is also notable that the E6 PBMs from the Group 1 HPV types have a statistically significant (P = 0.0083) increased preference for hScrib when compared with the E6 PBMs from the Group 2B HPV types (Fig 7B). Interestingly, the HPV-40 E6 PBM consistently failed to interact with hScrib in any of our assays (Figs 2 and 7A), and the full-length HPV-40 and HPV-66 E6 proteins, expressed as GST fusion proteins, also failed to bind to hScrib protein (Fig 7C). These results indicate that the ability of the high-risk HPV E6 oncoproteins to interact with hScrib correlates closely with their oncogenic potential.

### ZO-2 and β-2 syntrophin are novel HPV E6 interacting partners

The results from the proteomic analysis identified two potential novel interacting partners of certain high-risk HPV E6 proteins, the tight junction proteins, ZO-1/ZO-2, and the cell polarity regulator β-2 syntrophin (SNTB2). The detection of ZO-2 was particularly intriguing since recent studies have reported that it is stabilised by HPV in transgenic mice expressing the HPV-16 E6 oncoprotein [32].

**Fig 8:**
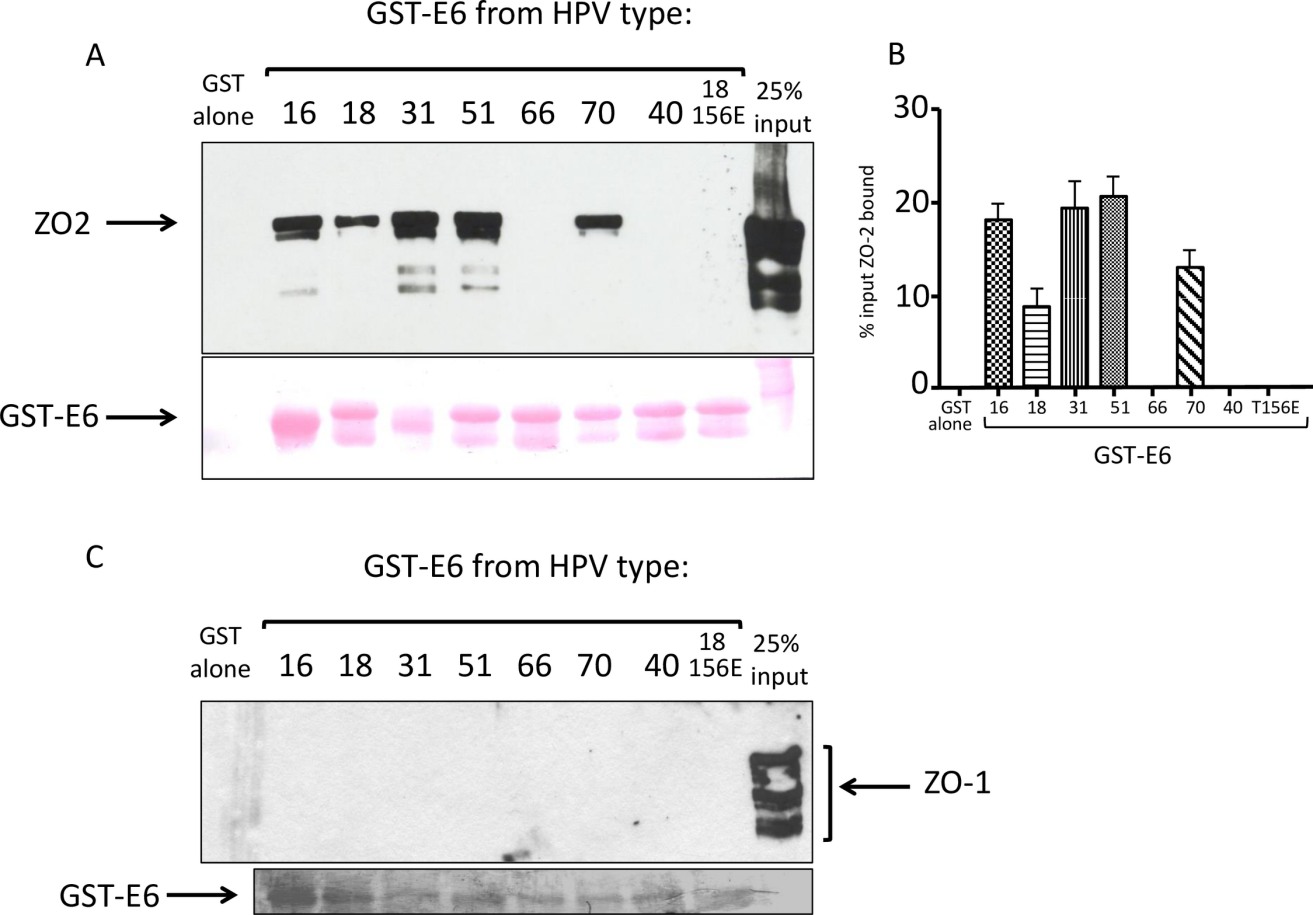
Novel interacting partners of HPV E6 proteins. ZO-2 binds to HPV E6 proteins in a PBM-dependent manner. **(A).** Extract of 293 cells transfected with ZO-2-expressing plasmid was incubated with GST-E6 proteins from HPV types 16, 18, 31, 51, 66, 70, 40 and the HPV18 T156E mutant, which ablates PDZ binding. Above: bound proteins detected by western blot probed with anti-HA antibody. Below: Ponceau staining of the nitrocellulose membranes, showing the GST-E6 proteins (arrowed). **(B).** The histogram shows the collated results of at least 3 assays. Standard deviations are shown. **(C).** No binding is seen with HA-ZO-1. Extract of 293 cells transfected with ZO-1-expressing plasmid was incubated with GST-E6 proteins from HPV types 16, 18, 31, 51, 66, 70, 40 and the HPV18 T156E mutant, which ablates PDZ binding. Above: bound proteins detected by western blot probed with anti-HA antibody. Below: Ponceau staining of the nitrocellulose membranes, showing the GST-E6 proteins (arrowed).

To confirm whether ZO-1/ZO-2 and SNTB2 are indeed novel interacting partners of certain high-risk HPV E6 proteins, we performed a series of pulldown assays using a range of GST-E6 fusion proteins incubated with an extract of HA-ZO-1 or HA-ZO-2-transfected 293 cells. After extensive washing the bound proteins were visualised by Western Blot probed with anti-HA antibody. The results in Fig 8 demonstrate a clear association between HA-ZO-2 and the E6 proteins from HPV types 16, 18, 31, 51 and 70, but not those from HPV types 66 or 40, which is consistent with the results from the mass spectroscopy analyses. No binding was seen with the GST-HPV18 E6 T156E mutant, which is null for PDZ binding, demonstrating that the interaction is, indeed, PBM-dependent. In contrast, ZO-1 does not bind significantly to any of the GST-E6 proteins tested, suggesting that the presence of ZO-1 in the pulldown screens was most likely as a result of its association with ZO-2.

**Fig 9:**
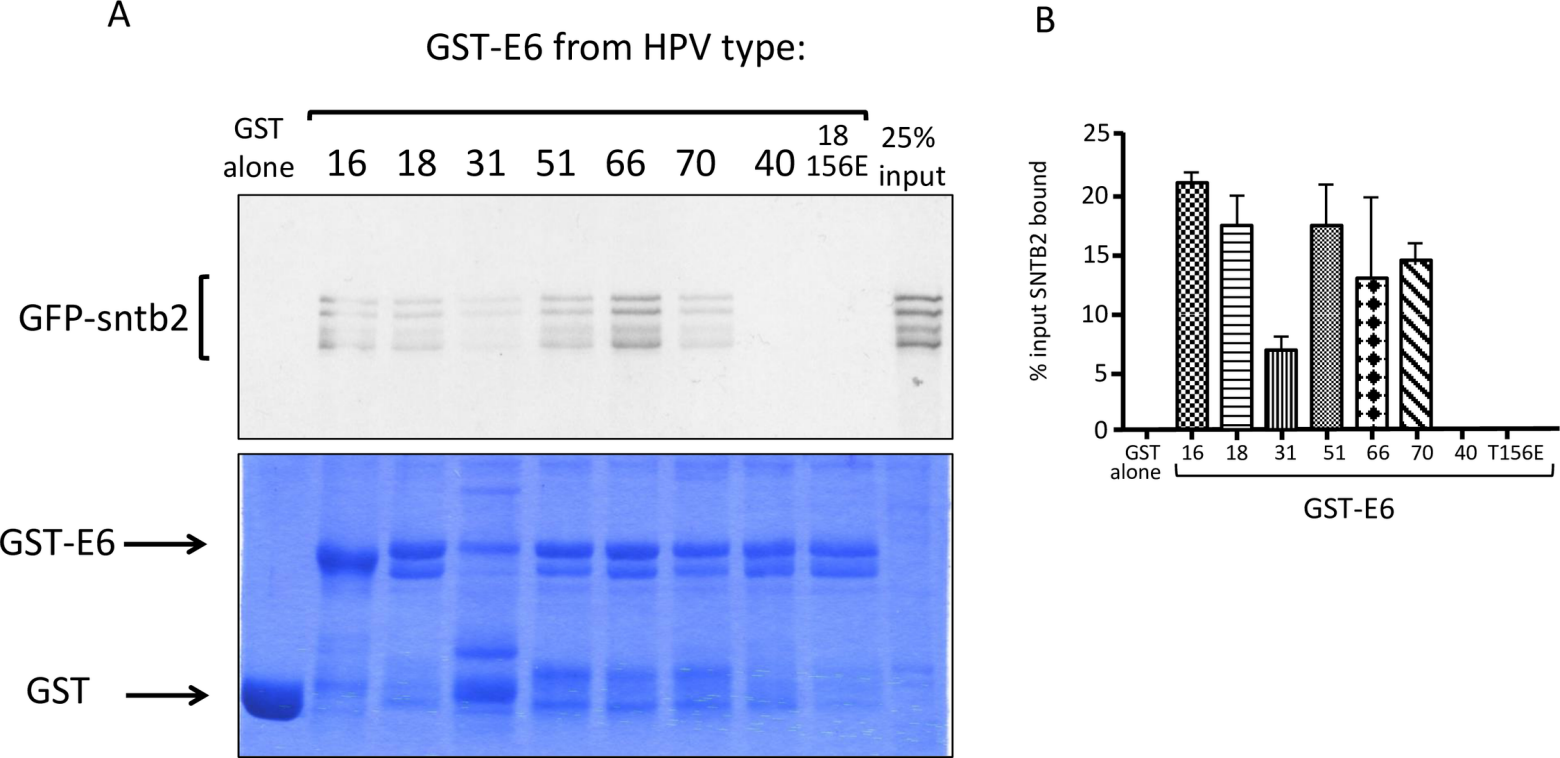
Novel interacting partners of HPV E6 proteins. Beta-2-syntrophin (SNTB2) binds to HPV E6 proteins in a PBM-dependent manner. **(A).** Autoradiograph of a GST pulldown assay using *in vitro* translated, radiolabelled SNTB2, together with GST-E6 fusion proteins from HPV types 16, 18, 31, 51, 66, 70, 40 and the 18E6T156E mutant. The Coomassie stained gel shows the GST fusion protein input (arrowed). **(B).** The histogram shows the collated results of at least 3 assays, standard deviations are shown.

Likewise, in vitro translated, radiolabelled SNTB2 (Fig 9) is efficiently bound by multiple high-risk HPV E6 oncoproteins in a PBM-dependent manner, with only HPV-40 E6 failing to bind, and again use of the GST-HPV18 E6 T156E mutant demonstrates that this interaction is PBM-dependent. Taken together these results indicate that ZO-2 and SNTB2 are novel PDZ domain-containing targets of the high-risk HPV E6 oncoproteins.

Although association with each protein in the mass spectroscopy analyses varied substantially between the C-terminal peptides of different HPV types, with HPV-70 for example being a particularly strong binder of ZO-2, there did not appear to be a significant correlation with the oncogenic potential of the different HPV types. However, it is noticeable that the majority of Group 1 E6 PBMs bind ZO-2, while other Group 2B E6 PBMs do not bind to ZO-2, and the ancestral HPV-40 E6 fails to recognise either ZO-2 or SNTB2 in these interaction assays.

**Fig 10:**
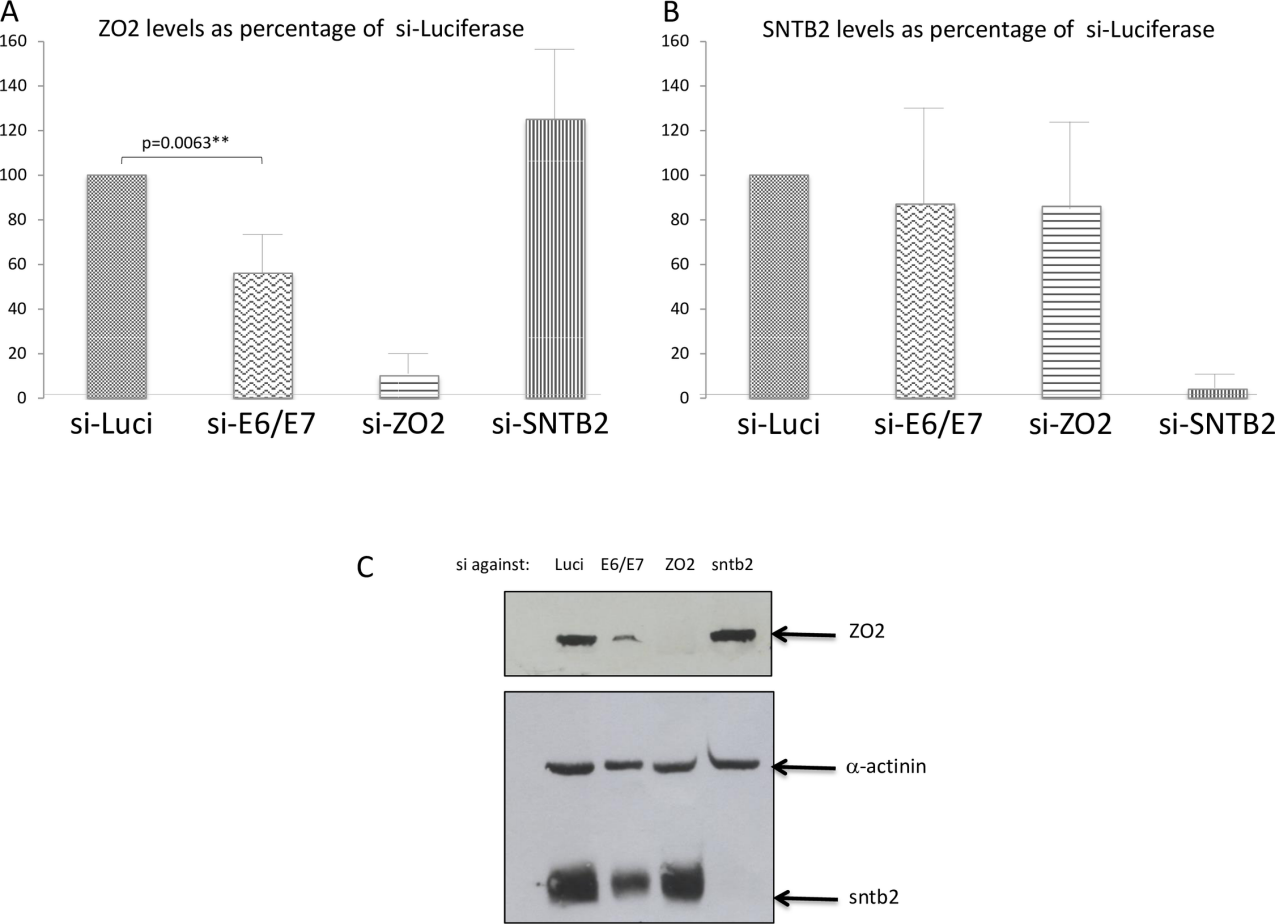
ZO-2 levels are increased upon HPV E6 expression. Western blot analysis of **(A).** ZO-2 and **(B).** SNTB2 levels in HeLa cells. si-ablation of E6/E7 expression results in a significant (p = 0.0063) reduction in ZO-2 levels. HeLa cells were transfected with siRNA to Luciferase (Luci) as control, with si-HPV-18 E6/E7 to ablate E6 expression, with si-ZO-2 or si-SNTB2. ZO-2 and SNTB2 levels were determined by Western Blot after 48h. The histograms show the results of at least three independent assays, analysed by Image J and Prism programmes and standard deviations are shown. **(C).** A representative western blot assay.

Having shown that the high-risk HPV E6 proteins can interact with ZO-2 and SNTB2, we were interested to know what might be the effect of this association. To investigate this, we treated HPV-18-containing HeLa cells with siRNA to Luciferase, HPV-18 E6/E7, ZO-2 or SNTB2. After 48h the levels of ZO-2 and SNTB2 in the cell extracts were analysed by Western Blot. Fig 10 shows a representative Western Blot, together with histograms showing the combined results of at least 3 assays. Clearly, the ablation of E6/E7 expression results in a significant reduction in ZO-2 levels, which is consistent with previous reports of E6 stabilising ZO-2 expression [32], whereas the levels of SNTB2 do not appear to be significantly affected, suggesting that the interaction with E6 has little effect upon the levels of SNTB2 protein.

**Fig 11:**
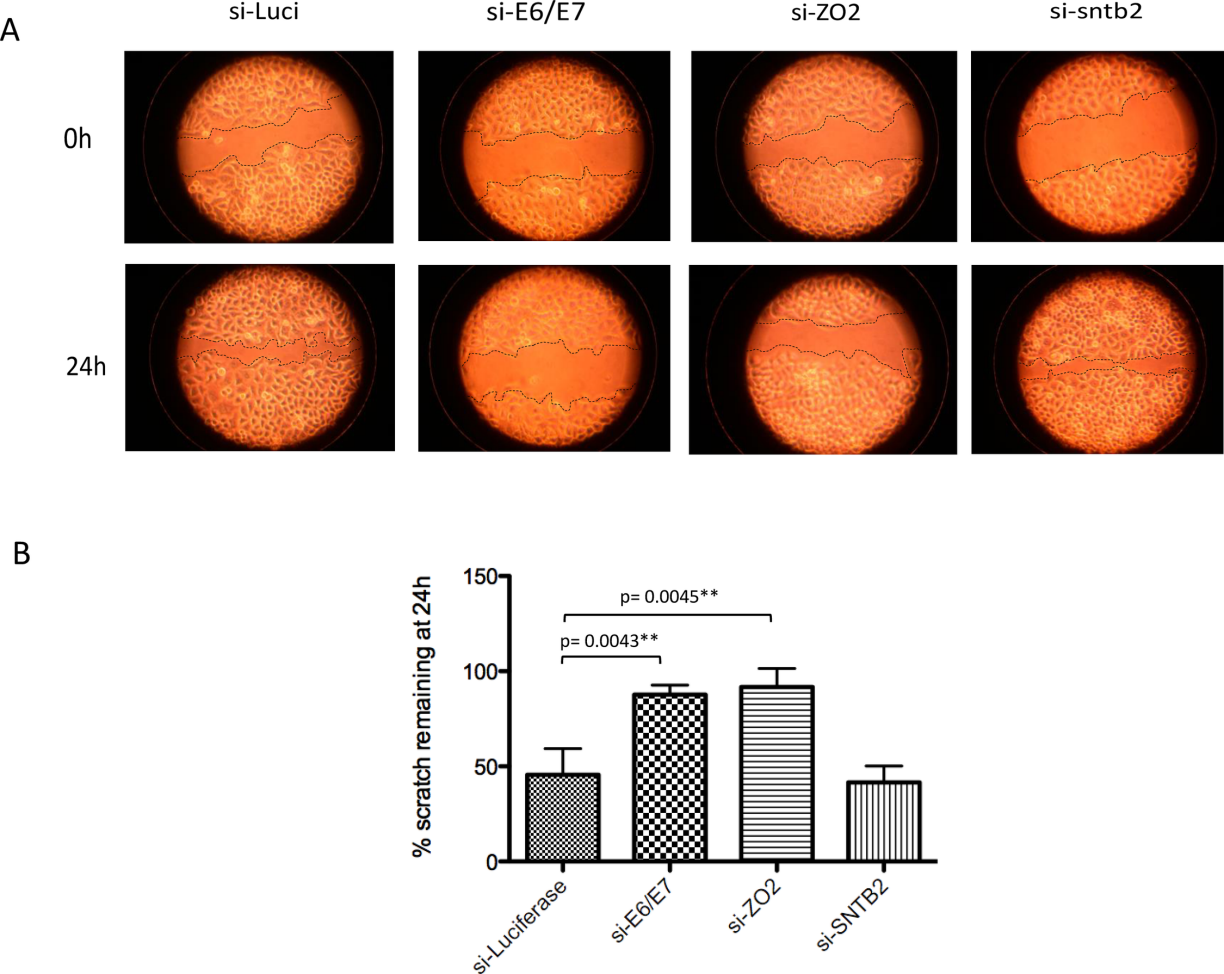
Increased ZO-2 levels promote cell migration. Ablation of ZO-2 expression reduces wound healing ability in HeLa cells, to a similar degree as ablation of E6 expression. **(A).** Confluent HeLa cells were scratched with a plastic pipette tip 48h after being transfected with si-RNA to luciferase, HPV 18 E6/E7, ZO-2 or SNTB2. The cells were photographed immediately post-scratch (0h) and after 24h. **(B).** The histogram shows the percentage area of the scratch remaining at 24h, from three independent experiments and standard deviations are shown.

Having confirmed that HPV E6 expression results in ZO-2 stabilisation we next asked what the biological consequence of this might be. To investigate this question we performed wound-healing assays in HeLa cells. The cells were transfected with siRNA as before and at 48h post-transfection the confluent cell sheet was scratched. The scratches were photographed immediately and then 24h later, and the area of scratch remaining was calculated. Fig 11 shows a representative assay plus a histogram of the collated results of at least three assays. It is clear that ablation of E6/E7 almost completely prevents wound healing, in comparison with si-Luciferase. Clearly knock-down of ZO-2 also has a similar inhibitory effect upon wound-healing, while ablation of SNTB2 has little or no effect.

These results indicate that loss of ZO-2 phenocopies loss of E6/E7 in these wound healing assays, and suggests that ZO-2 is a functionally relevant target of HPV-18 E6.

### Dissection of the molecular basis for substrate selection

**Fig 12:**
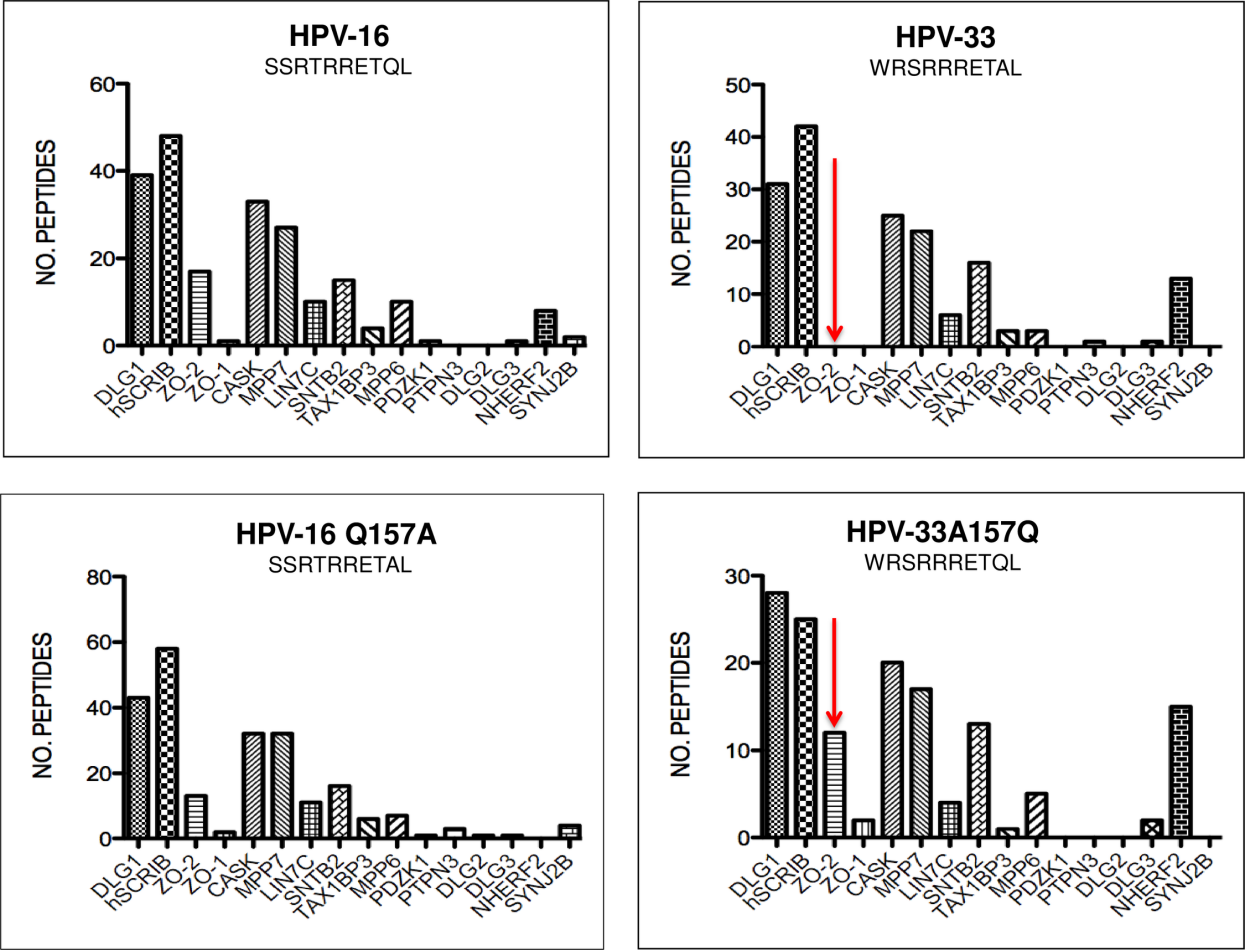
The molecular basis for substrate selection. Histograms representing the mass spectroscopy results of peptide pulldowns from HaCat cell extract, using peptides representing the C-termini of HPV-16 and HPV-33, and peptides in which the penultimate amino acid residue was swapped: HPV-16Q150A and HPV-33A148Q. The most marked effects of the change are arrowed. The histograms were drawn using the Prism software, the peptide sequences are shown on each histogram.

Since we had shown that the differences in protein sequences upstream of and within the PBM can influence target selection, we were interested in examining HPV-16 and HPV-33, which have the only E6 PBMs ending with Leucine, rather than Valine residues; and in HPV-16 this is known to increase the preference for binding hScrib [31]. The PDZ binding profiles of HPV-16 and HPV-33 are quite distinct, especially with respect to ZO-2, and comparison of their PBM sequences shows that HPV-16 has a penultimate Glutamine residue, while HPV-33 has Alanine at that position (Fig 1). To determine how this difference might affect the binding profiles we repeated the pulldowns using the HPV-16 and HPV-33 C-terminal peptides, together with the penultimate-swap peptides 16Q150A and 33A148Q. The histograms in Fig 12 show relatively minor changes in the binding profiles with respect to many of the target proteins, however ZO-2 binding is a major exception. The Q to A mutation in the 16E6 peptide gives a ~25% reduction in ZO-2 binding; conversely the A to Q mutation in the 33E6 peptides results in a large increase in ZO-2 binding. This is shown graphically in Fig 12, where it can be seen that the amino acid swap has coordinate effects upon the target binding; thus DLG1, hScrib, ZO2, Lin7c and SNTB2 interactions are inversely affected by the change A to Q or Q to A. This underlines the importance of individual non-canonical residues of the PBM in target binding specificity.

## Discussion

In this study we have performed a systematic analysis, in two different keratinocyte cell-lines, of the substrate specificities of the E6 PBMs derived from multiple high-risk HPV types and confirmed at least 19 different PDZ domain-containing proteins as potential interacting partners of the HPV E6 oncoproteins. Previous studies had investigated the PDZ binding profiles of the HPV-16 or HPV-18 E6 PBMs [21,22], but this is the first comparative analysis of the substrate recognition profiles of a range of cancer-associated and non-cancer-associated HPV types. This study demonstrates some major differences in the potential PDZ interaction profiles of different HPV E6s, highlighting intriguing aspects of the profiles that are conserved across multiple E6 proteins, whereas certain other E6-interaction profiles are associated with particular oncogenic HPV types.

Whilst our study and previous analyses indicate a large number of potential PDZ interacting targets of the HPV E6 PBMs, the actual number of PDZ substrates bound by any specific HPV E6 PBM is generally much lower. This number varies depending on the specific HPV type, with, for example, the PBMs of highly oncogenic HPV-16 and HPV-18 E6 being capable of recognising the most substrates at any one time, whilst in contrast, the perfect consensus PBM of HPV-66, interacts with many fewer PDZ substrates. In fact, a very clear trend was observed throughout this analysis, where the E6 PBMs from the most commonly cancer-associated HPV types bound significantly (P = 0.0029) more PDZ substrates than the HPV E6 PBMs from types less frequently, or never, associated with human tumours. This suggests that an important feature of the E6 PBM is not just its capacity to interact with a particular cellular PDZ domain- containing protein, but also the functional diversity of the PBM, where there is a higher degree of functional flexibility in the cancer-causing HPV E6 oncoproteins.

Throughout this study we did not detect any major differences between the Group 2B and the Group 3 viruses, although HPV-70 had a slightly broader interaction profile and does appear to have a marked ability to recognise ZO-2. This is intriguing as recent studies have reported a low incidence of HPV-70 in single-infection cervical cancers [33], and further studies are required to investigate this further.

Analysis of the specific substrates bound by the different HPV E6 PBMs emphasises the overall commonality in interactions with hDlg, with all the types tested showing a very robust association. This result was particularly surprising for the non-oncogenic HPV-40 E6 of the α8 genus, which has recently been shown to possess an ancestral PBM [25]. Despite this E6 protein having a very different PBM from the other types analysed, it still bound hDlg and pulled down its associated CASK/MPP7/Lin7c complex in a similar manner to the canonical E6 PBMs of HPV-16 and HPV-18. This suggests that the capacity for binding to hDlg, whilst probably being *necessary* to confer an oncogenic phenotype upon an E6 protein, is not, alone, *sufficient* to do so. Intriguingly, HPV-40 E6 demonstrated minimal levels of association with other PDZ domain-containing substrates, suggesting that this ancestral PBM has much less functional flexibility than the more well-known high risk HPV E6 PBMs. The recent study of van Doorslaer et al [25] suggests that the development of a PBM allowed primitive HPVs to colonise a new niche, but that the ability to bind PDZ domains was a two-edged sword for the virus, since survival in the new niche required the development of additional means of interfering with the cellular environment, with the consequent risk of oncogenic transformation of the host cell. This suggests that acquisition of an ability to interact with hDlg could have been instrumental in facilitating the occupation of a new niche upon the appearance of a PBM on E6, and furthermore, that this has retained functional relevance for the virus life cycle throughout the evolution of these HPV types.

In contrast to their interactions with hDlg, the different HPV E6 PBMs displayed striking differences in their capacities to interact with hScrib. In agreement with previous studies, hScrib was bound strongly by HPV-16 and HPV-18 E6, and was pulled down by PBMs of all the cancer-causing category I HPV types 31, 33, 35, 51 and 56. Of these, HPV-56 interactions with hScrib are weak, but nonetheless consistent. It should be noted however that its association with cervical cancer is also very rare. In support of this, E6 PBMs from types less frequently associated with cancer bound hScrib either not at all or much more weakly (P = 0.0083), suggesting that interaction with hScrib is a good predictor of oncogenic potential. This correlates well with data showing that knockdown of hScrib in keratinocytes can decrease their cell-cell junction formation and increase their invasive potential [34]. In addition, recent studies have highlighted a potential critical role for hScrib in a number of models of tumour development through Ras/ERK/MAPK signalling [35,36,37]. Clearly, mislocalisation of Scrib in a murine model of breast cancer has potent oncogenic activity [38] that appears to be related to the control of mTOR signalling [39], and this also occurs in HPV-positive cells [40].

Two novel PDZ domain-containing targets of E6 were identified in the course of this analysis, the ZO-1/ZO-2 complex and β-2 syntrophin (SNTB2). A recent study had indicated that the tight junction component ZO-2 was overexpressed in transgenic mice expressing HPV-16 E6 [32], and we show here that ablation of HPV-18 E6 expression in HeLa cells also results in lower levels of ZO-2 protein. It thus appears that ZO-2 stabilisation is a common feature of HPV16 and HPV-18 E6s, and since we show here that ZO-2 is bound by all of the Group 1 HPV E6 proteins analysed, it seems likely that these interactions may also result in higher levels of ZO-2 within the cell. In addition, our finding that ZO-2 ablation will inhibit wound healing, even in the presence of the E6 and E7 oncoproteins, suggests that at least some of the migration-promoting potential of E6 may be mediated through ZO-2.

SNTB2 is a component of Dystrophin complexes, found at the inner surface of plasma membranes, in which it is thought to regulate membrane stability and to provide a scaffold for the assembly of multiprotein signalling complexes. Previous reports had also suggested that the HPV-16 and HPV-18 PBMs could recognise SNTB2 [21,22]. In our analysis SNTB2 was bound by all the E6 types tested, with the exception of HPV-35 E6. There were residual levels of association with the ancestral PBM of HPV-40 E6, with varying degrees of interaction with the E6 PBMs of other HPV types.

In agreement with previous studies [23,24], very minor alterations to the residues within the PBM confirm the critical contribution of non-canonical residues to PDZ substrate selection. This was exemplified perfectly with HPV-33 and HPV-16 E6, which have very similar interaction profiles and very similar PBMs, except that HPV-16 E6 bound ZO-2 much more efficiently than HPV-33. By swapping A/Q residues within the E6 PBMs we effectively swapped their respective capacities to recognise ZO-2.

Taken together this study presents an intriguing picture of how variations in the HPV E6 PBM influence PDZ substrate selection. We present compelling evidence of the acquisition of enhanced functional flexibility in the cancer-causing HPV E6 oncoproteins, and identify hDlg as an evolutionarily conserved target of all the HPV E6 PBMs analysed. In contrast, acquisition of an ability to interact with hScrib correlates closely with increased cancer-causing potential.

## Materials and Methods

### Peptides

The sequences of the biotinylated peptides that were synthesised in-house are shown in Fig 1.

### Cells and transfection

The spontaneously immortalised keratinocyte HaCaT cell line [41] was kindly provided by Dr Massimo Tommasino and was grown in Dulbecco's modified Eagle's Medium (DMEM) supplemented with 10% foetal calf serum, penicillin/streptomycin (100U/ml) and glutamine (300μg/ml).

HEK293 cells (ATCC) were grown on 10cm dishes in the same medium and were transfected by the calcium phosphate precipitation method [42].

HeLa cells (ATCC) were grown on 6-well plates in the same medium and were transfected with siRNAs using the Invitrogen RNAimax reagent.

The Normal Immortal Keratinocytes (NIKS) cells [43,44] were kindly provided by John Doorbar and were grown in F medium (3:1[v/v] F12:DMEM media, supplemented with 5% foetal calf serum, 0.4ug/ml hydrocortisone, 5ug/ml insulin, 8.4 ng/ml cholera toxin, 10ng/ml EGF, 24ng/ml adenine, 100U/ml penicillin/streptomycin.

### Cell extraction for mass spectroscopy

Soluble proteins were extracted from 80% confluent HaCaT or NIKS cells by incubation for 10min on ice in lysis buffer (50mM HEPES pH7.4, 150mM NaCl, 1mM MgCl_2_, 1% Triton-x-100, protease inhibitor cocktail I [Calbiochem]). The cells were removed from the plate by scraping and the debris removed by centrifugation at 14000rpm in a benchtop centrifuge for 2min at 4°C.

### Peptide pulldown

500μg of each peptide, in lysis buffer, was bound to streptavidin-conjugated magnetic sepharose beads (Streptavidin-MagSepharose, GE Healthcare) by incubation at 4°C on a rotating wheel for 1h, then washed three times with lysis buffer.

The cell extract was pre-cleared by incubation with empty streptavidin conjugated magnetic beads at 4°C on a rotating wheel for 1h.

After removal of the pre-clearing beads, the extract was incubated at 4°C on a rotating wheel for a further 2h with each of the biotinylated peptides bound to streptavidin-conjugated magnetic sepharose beads.

The beads were washed three times with lysis buffer without protease inhibitors, transferred to fresh eppendorf tubes and washed twice more with lysis buffer without either protease inhibitors or Triton-x-100.

5% of the beads were taken for western blot analysis and the remainder were subjected to trypsin-digest and the products analysed by mass spectroscopy, as described previously [45].

### GST binding assays

The cloning and use of the GST Dlg1 fusion proteins have been described previously [28]. The GST-E6 proteins from HPV types 16, 18, 31, and 51, plus HPV-18 T156E mutant have been described previously [23], The HPV-40, HPV-66 and HPV-70 E6s were synthesised by the GeneArt Gene Synthesis protocol (Invitrogen) and cloned into the BamHI/EcoRI sites of the pGEX2T and pCA plasmids for GST fusion protein expression and in vitro/in vivo expression, respectively. The Dlg1, hScrib and SNTB2 proteins, plus the HPV-18 and HPV-40 E6 proteins, were expressed in vitro using the Promega TnT kit and the ZO-1 and ZO-2 proteins were expressed by transfecting the plasmids into HEK293 cells. The pCDNA plasmids expressing Dlg1 and hScrib have been described previously [28,31]. The pGWI:HA-ZO-1 and ZO-2 plasmids were the kind gift of Ron Javier, and the SNTB2 expressing plasmid was the gift of Marvin Adams.

### siRNA

The following siRNAs were purchased from Dharmacon: si-Luciferase, si-HPV18 E6/E7, si-ZO-2, si-SNTB2, and were transfected using the RNAimax reagent (Invitrogen) according to the manufacturer's instructions.

### Scratch assays

HeLa cells were transfected with siRNA, as indicated, and after 48h the confluent cells were scratched with a sterile Artline p2 pipette tip (Thermo Scientific). The cells were washed twice with PBS to remove any loose cells and immediately photographed using a Nikon COOLPIX995 camera. After 24h incubation the scratches were photographed again and the cell-free area quantified using the Image J and Prism programmes. The cells were then harvested in lysis buffer and the ZO-2 and SNTB2 levels analysed by western blot. Alpha actinin was used as a control for total cellular protein levels.

### Antibodies

The following antibodies were used: Mouse anti-DLG1 (Santa Cruz); goat anti-hScrib (Santa Cruz); rabbit anti-ZO-2 (Cell Signaling); mouse anti-SNTB2 (Pierce); mouse anti-HA (GE Healthcare); goat anti-CASK (Santa Cruz).

Appropriate HRP-coupled secondary antibodies were purchased from DAKO.

## Supporting Information

S1 Fig The mean numbers of cellular protein peptides detected by the mass spectroscopy to be bound by each E6 C-terminal peptide were normalised with respect to the numbers of DLG1 peptides bound, such that DLG1 = 100. No material differences in the profiles were seen compared with those shown in Fig 1B.
(TIF)
