# Supplementary figures and images for: Analysis of Multiple HPV E6 PDZ Interactions Defines Type-Specific PDZ Fingerprints That Predict Oncogenic Potential
Source: PLoS Pathog. 2016 Aug 2;12(8):e1005766. doi: 10.1371/journal.ppat.1005766 (PMC4970744; doi:10.1371/journal.ppat.1005766)

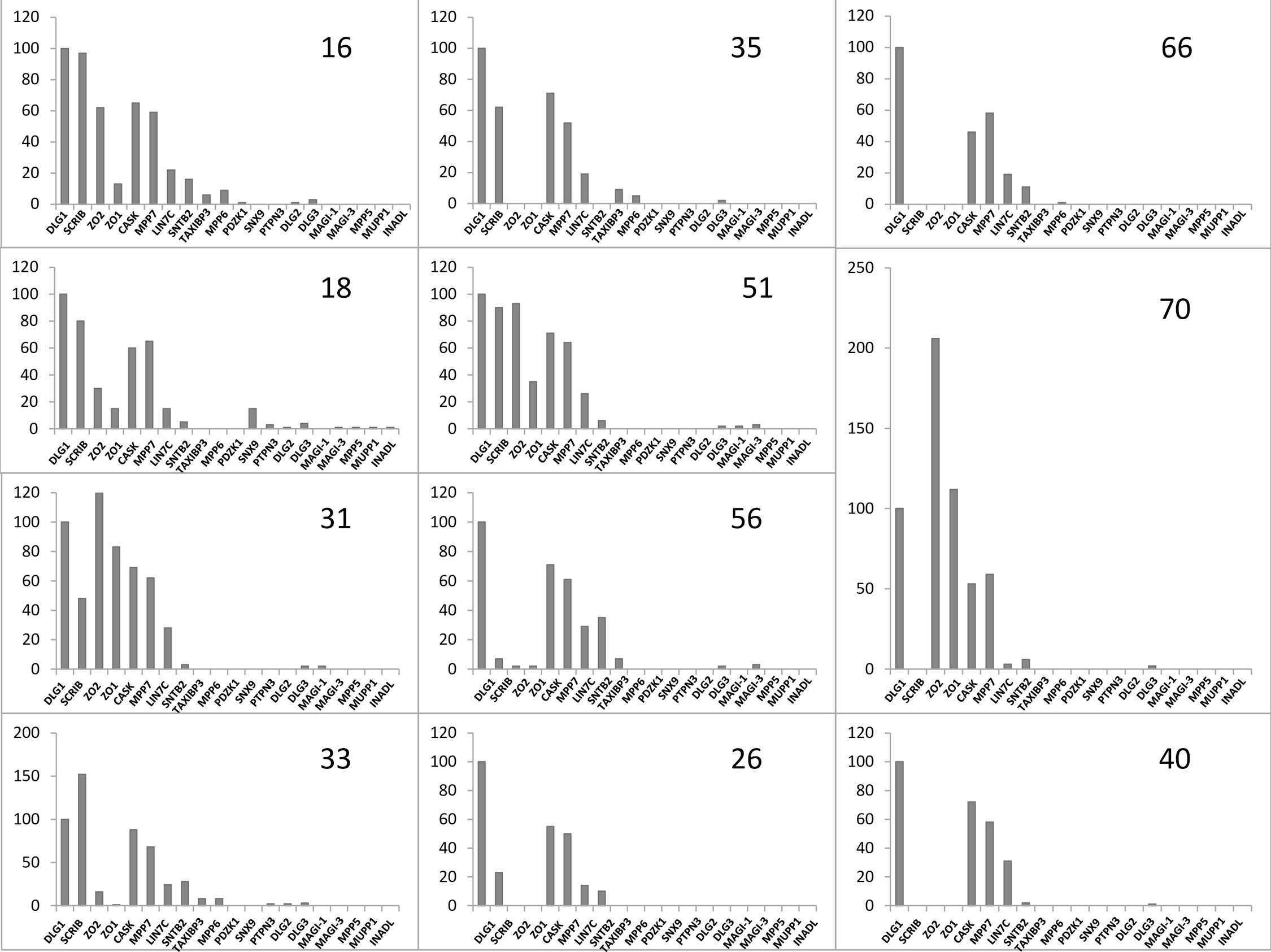

Supplement: S1 Fig — No material differences in the profiles were seen compared with those shown in Fig 1B. (TIF) [file ppat.1005766.s001.tif]
